# Unveiling the genetic diversity in horsegram (*Macrotyloma uniflorum* L.) genotypes through morphological and microsatellite (SSR) markers

**DOI:** 10.1038/s41598-025-10387-2

**Published:** 2025-10-07

**Authors:** Ragini Padha, Sanjeev Kumar, Radheshyam Kumawat

**Affiliations:** 1 Division of Plant Breeding and Genetics, Sher-e-Kashmir University of Agricultural Sciences and Technology, Jammu, 180009 J&K India; 2 School of Crop Improvement, College of Post Graduate Studies in Agricultural Sciences, Central Agricultural University (Imphal) Umiam, 793103 Meghalaya, India

**Keywords:** Horsegram, Variability parameters, Correlation, SSR, Polymorphism, Genetics, Plant sciences

## Abstract

Horsegram (*Macrotyloma uniflorum* L.) is a climate-resilient legume crop with significant nutritional value, yet its genetic potential remains underutilised. Understanding the genetic diversity in advanced breeding lines of horsegram (*Macrotyloma uniflorum* L.) can aid in developing effective selection method for seed yield improvement. This study evaluates the genetic variability and diversity of 22 advanced horsegram breeding lines using both morphological traits and molecular markers (SSR) to enhance yield and adaptability. Eleven morphological traits, including Plant height, No. of primary branches, Days to 50% flowering and maturity duration, Harvest Index, and Seed yield per plant, were analyzed to estimate genetic variability. Heritability and genetic advance percentage of mean were calculated to identify effective selection traits at early stage. Additionally, 30 SSR primers were screened to analyze molecular diversity, of which 9 polymorphic primers were identified. Cluster analysis, dendrogram, and Principal Component Analysis (PCA) were performed to group lines based on both morphological and molecular data. Significant variability was observed across morphological traits, with 1000-seed weight exhibiting high heritability and genetic advance, making it a promising selection trait. positively correlated with most traits except PH and primary branches per plant. Cluster analysis grouped the 22 lines into four morphological clusters, with HPKM-150 identified as the most diverse and high-yielding line. Molecular analysis revealed two main genetic clusters, with the primer MUMS-18 showing the highest polymorphic information content (PIC = 0.70). The study emphasizes significant genetic variability in horsegram, highlighting valuable traits and SSR markers for diversity assessment and future breeding to improve yield and genetics.

## Introduction

*Macrotyloma uniflorum* (Lam.) Verdcourt, commonly known as horsegram, is modern India’s 5th most widely grown legume^1^. It is a strictly self-pollinated crop species belonging to the family *Fabaceae* and having chromosome number 2n = 20, 22, and 24^2^. The genome size of the horsegram is relatively small, measuring approximately 398 megabases (Mb) in size^3^. It is widely presumed that India is the centre of origin for cultivated species of horsegram^4^. Wild horsegram has been reported in regions across Africa (Central, East, and Southern Africa) and India^5,6^. As a result, Southwest India^7^ and Africa^8^ are regarded as gene-rich centres of horsegram^9^. In India, it is mainly cultivated for food and fodder purposes in the southern states such as Karnataka, Andhra Pradesh, Tamil Nadu, Odisha, Maharashtra and Chhattisgarh as both *rabi* and *kharif* crops and the foothills of Himachal Pradesh, Jammu and Kashmir and Uttrakhand as *kharif* crop^10^. India covers an area of about 348 Mha with a production of 226 MT and productivity of 650 kg/ hectare. In J&K, horsegram is grown on an area of 0.71 Mha with a production of 0.24 MT and productivity of 338 kg/ hectare^11^.

Horsegram is a drought-hardy crop suited for cultivation in regions with sub-humid to semi-arid climates at elevations of up to 1800 masl. It thrives in temperatures ranging from 25^o^ to 30^o^C, withstanding temperatures up to 40^o^C. However, it is entirely intolerant to frost, excessive moisture or water-logged conditions^12^. It is highly suitable for rainfed and marginal agricultural land where other crops do not thrive and invariably fail to survive^13^. The minute natural seeds of horsegram offer remarkable benefits for holistic health, given their high concentration of essential nutrients such as protein, carbohydrates, and essential amino acids and are a good energy source^14^. It contains protein 17.9–25.3%, carbohydrates 51.9–60.9%, essential amino acids, and a low content of lipids 0.58–2.06%^15^. Thus, it can be used for pacifying various nutrient deficiencies as it contains molybdenum, phosphorus, iron and vitamins such as carotene, thiamine, riboflavin, niacin and vitamin C^16^. Consequently, horsegram has garnered attention as a prospective future crop species for improving the nutritional security of rural, tribal and underprivileged people^17^. Moreover, horsegram showcases numerous therapeutic attributes, including its potential as an antioxidant and antimicrobial agent, and it has demonstrated efficacy in the dissolution and displacement of renal calculi. Also, it helps in the dietary management of obesity due to the presence of beneficial bioactive compounds, namely phenolics and phytic acid^18^.

In spite of all these established facts, this crop has been neglected within the realm of mainstream agriculture in developing nations such as India, where only a few conventional legumes dominate pulse production^19^. This may be due to the non-availability of improved and well-adapted varieties. However, the recent trends seem to reverse. To address this challenge, introgression of desirable characteristics from both the cultivated and wild species in well-suited genotypes is essential. Prior to commencing the horsegram improvement programme, it is essential to comprehensively assess the genetic diversity within the existing germplasm. In horsegram, limited investigations on morphological, biochemical and molecular diversity have been reported^4,20^). However, there has been no concurrent comparative investigation conducted at both the morphological and molecular levels to evaluate the genetic diversity, particularly in the Jammu region of UT of J&K. Hence, the current research was undertaken to evaluate the genetic diversity within the existing horsegram germplasm; employing field-level morphological descriptors and molecular-level SSR primers.

## Materials and methods

### Field evaluation

A total of 22 advanced breeding lines of horsegram were included in the study (Table 1). The field experiment was conducted at the experimental farm of Pulses Research Sub-Station (PRSS), Samba, SKUAST-Jammu during *kharif* 2022 in randomised complete block design with three replications in which each genotype was planted in 2 rows, each of 3-meter in length. Row-to-row and plant-to-plant spacing were maintained at 45 × 10 cm^2^, respectively. From each plot, ten plants were selected at random to record observations on 11 yield contributing traits, which include vegetative and flowering traits. Genetic parameters, namely genotypic and phenotypic coefficient of variation (GCV & PCV), were estimated for each character as per the formula put forth by^21^ and were categorised as low: values lower than 10%, moderate: 10–20% and high: values greater than 20%. The genetic advance was calculated using the formula outlined by^22^. Heritability (h^2^) was estimated in the broad sense as per the formula proposed by^23,24^). Other genetic parameters such as correlation analysis^25^, and genetic divergence analysis^26^ were also conducted following the procedure of the authors given.

### DNA extraction

Seeds of all the 22 genotypes / lines were grown in pots in the glass house. The fresh young leaves were taken for the isolation of DNA following the CTAB method^27^ with some modifications in the Central Laboratory of Institute of Biotechnology, SKUAST- Jammu. The quality and concentration of the extracted DNA were estimated on 0.8% agarose gel before using in PCR reactions.

### SSR markers for PCR amplification

30 SSR molecular markers were selected using the insights from previous studies (Table 2) to amplify horsegram genomic DNA. Each PCR mixture consisted of 1.0 µL DNA (50ng/ µL), 3.0 µL PCR buffer (1X), 0.35 µL dNTPs (0.25mM), 1.2 µL MgCl_2_ (2.0mM), and 1.0 µL primer (10 µM / µL), 0.15 µL *Taq* DNA polymerase (0.75 U/ µL) and rest deionised water to make a final volume of 15 µL. The PCR cycles commenced with an initial denaturation phase at 94 °C for 4 min, and the amplification process underwent 37 cycles. Each cycle encompassed denaturation at 94 °C for 30 s, followed by annealing within a temperature spectrum of 50 °C to 62 °C for 30 s, and extension at 72 °C for 30 s. A culminating extension step was then implemented at 72 °C for 5 min. (Note: The annealing temperatures were standardized independently for each primer). The resultant amplified PCR product was subsequently preserved at 4 °C. PCR products were first checked on a 3% agarose gel and then resolved in 6% polyacrylamide gel at a constant current of 65 W at room temperature for 90 min. Gels were prepared and run in 1× TBE buffer, and fragments were visualised using silver staining. Size estimation of the alleles was done by using a 100 bp DNA size standard.

### Statistical analysis

The morphological data for all 11 traits were analysed for mean, GCV, PCV, heritability, genetic advance, and correlation analysis using the statistical software R Studio Version 1.4.1564. Genetic divergence analysis involved D^2^ statistic, which was performed using the software Windostat 9.3. The molecular data was analysed using NTSYS 2.0 software, employing binomial data representation. Based on DNA amplification results, SSR bands were manually assessed and coded as 1 for presence and 0 for absence. Distance-based cluster analysis was performed by generating dendrogram based on unweighted pair-group method of arithmetic mean (UPGMA). Principal component analysis (PCA) was performed using Past 3 software. The polymorphism information content (PIC) of each primer pair was calculated according to the formula given by^28^.

**Table 1:** Details of promising advanced breeding lines of horsegram used in this study.

SI. No.	Name of genotypes	Source	Pedigree
1.	AK-21	IGKV, Raipur	–
2.	Bilas-9	IGKV, Raipur	Local land race of Bilaspur district of Chhattisgarh
3.	BSP-17-1	IGKV, Raipur	Local land race of Bilaspur district of Chhattisgarh
4.	BSP-17-3	IGKV, Raipur	Local land race of Kobra district of Chhattisgarh
5.	BSP-19-2	IGKV, Raipur	Local land race of Baster district of Chhattisgarh
6.	BSP-19-2	IGKV, Raipur	Local land race of Pendra district of Chhattisgarh
7.	HPKC-02	CSKHPKV, Palampur	Local Collection from Palampur, District Kangra
8.	HPKC-11-01	CSKHPKV, Palampur	(HPK-3 × HPK-4) × (VLG-1 × HPKC-7)
9.	HPKC-11-04	CSKHPKV, Palampur	(HPK-3 × HPK-4) × (VLG-1 × HPKC-7)
10.	HPKC-11-17	CSKHPKV, Palampur	(VLG-1 × HPKC-7) × (HPKC-3 × HPC-4)
11.	HPKC-11-25	CSKHPKV, Palampur	(VLG-1 × HPKC-7) × (HPKC-3 × HPC-4)
12.	HPKC-11-47	CSKHPKV, Palampur	(VLG-1 × HPKC 3) × (HPKC-3 × HPKC-4)
13.	HPKC-11-56	CSKHPKV, Palampur	(HPKC-3 × HPK-4) × (VLG-1 × AK-21)
14.	HPKC-11-57	CSKHPKV, Palampur	(HPKC-3 × HPK-4) × (VLG-1 × AK-21)
15.	HPKC-11-74	CSKHPKV, Palampur	VLG-1 × AK-21
16	HPKC-4	CSKHPKV, Palampur	Mutant of HPKC-02
17.	HPKM-150	CSKHPKV, Palampur	Released variety of CSK HPKV, Palampur
18.	MC-5-3	CSKHPKV, Palampur	(HPKM191 XHPK4) × Himaganga
19.	MC-6-4	CSKHPKV, Palampur	(HPKM317 × HPK4) × (HPKM191 × HPK4)
20.	MC-6-5	CSKHPKV, Palampur	(HPKM317 × HPK4) × (HPKM191 × HPK4)
21.	VLG-15	CSKHPKV, Palampur	VL Gahat 1 × NIC 7321
22.	Thaira kulthi-2	IGKV, Raipur	–

**Table 2:** Details of the SSR markers including their annealing temperatures used in the present study.

S. No.	Primer	Primer sequence	Annealing temperature Ta (°C)	Amplicon size (bp)
1.	HUGMS-12	F-GAGCACAAAGAAGGGTTCGT R-TGCAAATCACAAGAAGGAAAGA	52°	650 − 600
2.	HUGMS-15	F-ATCGGCACGAGGATTAGGTC R-TGGTTCAAAACCCCATAATCTC	52°	140–160
3.	HUGMS-25	F-GGTTGCATAATGTGGTGCAG R-CAGCAGCAGTAGCCACAAAG	53°	170
4.	MUMS-11	F-TGGGAAAGAAAAGACAAACGA R-TGATGCTTCAATTATGGCTTTG	49°	180
5.	MUMS-18	F-TCCCTTTCCGTTTCAACAAG R-GAGAATGATGATGCCAACGA	49°	180–230
6.	MUMS-19	F-CCAGCGAAGACACAAGACAA R-GTTCGGTGAAAGCAAGAAGC	53°	150–210
7.	MUMS-24	F-GCAGGGAAAGAATTTGTTGG R-CCACAATAGCAGCAGCAGAA	50°	450–460
8.	MUMS-26	F-AGCTCAACCACAAGAATTATAGGT R-CCCTCATCTACTGGGATTTGG	52°	230
9.	MUMS-30	F-CTGTGGAGCGACCTAAGAGG R-AAGTTTCCAGCACCCTTCCT	54°	210
10.	MUMS-35	F-ACCAACCAAACCATTGGGTA R-TTTCTTCGAGGTCCATGTCC	52°	205
11.	MUMST-21	F-CGATGCCTCGTGTTTGTTAC R-GGTGCCTCCTTTACCCATTT	52°	320–330
12.	HTSSR155	F-TACAATTTCCTCCAAACCAG R-GAAGAAGACATGGCCAGTTA	49°	–
13.	HTSSR151	F-AAGGGTTAGGGTTCATGATT R-CTGCACCATCCACAAAAC	49°	–
14.	HTSSR105	F-GCCATAAGCTGTGAAAGAGA R-TAAAATCAGAACCAGCGAGT	49°	–
15.	HTSSR 21	F-CAGAGTTTGCCACTGTTACC R-AAAAGCACTCAAGAAGTCCA	49°	–
16.	HTSSR 104	F-GGCTCCAAGTCCTAATCAAT R-TAGCGAACTCACCAATTTTC	49°	–
17.	HTSSR153	F-TGAAGAAGGAAGATGAAGGA R-AGAGATTGCATTGCTATGGT	49°	–
18.	MUMS 02	F-CCCCAGAAATTCCTTGCAT R-TTCGCGGTAAATCTGGCTAC	52°	200–260
19.	MUMS 10	F-CCCAAATATCGTGGAATTGG R-CCTGGCTTCCACACTGTTCT	52°	470–510
20.	MUMS 7	F-TGGTGGATTGAATATTGAAGGA R-TTCAACCCAACACCAACACA	50°	295–380
21.	MUMST 12	F-CCAAGACAAGACCACACTGC R-CCATGAACCACAGCATGTTC	53°	200–240
22.	HUGMS17	F-TCCCTCTTGTGGCTGGTATT R-TGCAGCTAGCTAGGGCTGAT	55°	240–280
23.	HUGMS 26	F-CACGAGGGTGATGAAGATGA R-ATCCTCAAACATGCCCTCAC	52°	150–170
24.	MUMSD-21	F-GCACCCACATTCCACGATAC R-AAAGCGAGGAGGTGAGTTGA	54°	140–160
25.	MUMSD-05	F-GGCATCTGAGAGGCAGAGAA R-CGCCAACCAGTGAGAAAGTT	54°	180–210
26.	HUGMS-05	F-CACTTTCGATTTGGGTTTCC R-CCTCAAAGTCCAAGCCAGAC	50°	240–250
27.	HUGMS-33	F-TAAACGCCACTGCATTGAAC R-TTACCTTTCCCTCCCCAAGT	52°	210–235
28.	MUMST-40	F-GTTCCCTCTTCCCCTTCATC R-ATTTCACCGGCTTCTCTTCA	52°	245–255
29.	MUMST-26	F-TCAGTGGCATTTCATCAAGC R-TTCTTCTTCCCCATCGATCA	50°	250–255
30.	MUMST-49	F-GATTTCGTCGAGCGAGTACG R-TCCACATTCGATCCTTCCTC	52°	135–140

## Results and discussion

### Morphological analysis

Analysis of variance revealed that significant variation exists among the different genotypes included in the study for all the characters considered, indicating that the material selected was quite variable (Table 3). The measures of PCV, GCV, heritability (broad sense), and genetic advance (GA) for yield and its contributing characters are represented in Table 4. The study revealed that the PCV was higher than the GCV for all the characteristics examined, indicating a significant genotype × environment interaction^20,29^). Traits such as 1000SW^30^, CP (Alle et al., 2015), NPP, and SY^31^ showed high PCV and GCV values; PH and DTF^20^, PL^32^, and HI^33^ exhibited moderate values; while DTM^32,34^) and SP^35^ revealed lower values for PCV and GCV. These traits were all reported in horsegram, suggesting that traits with lower variation are more influenced by environmental factors and may respond less to phenotypic selection.

### Genetic parameters

Heritability determines how likely a character is to be inherited across generations. In the present study, all the characters’ heritability estimates ranged from moderate to high (Table 4). Characteristics like 1000SW, SY, NPP, CP, PL, SP, and DTM exhibited significant heritability^20,31,36^). Moderate heritability estimates were observed for traits such as the PB, PH, and HI^29^, and for DTF in blackgram^37^.

Genetic advance serves as a valuable indicator of the anticipated progress in effective and efficient selection when applied to the base population^38^. When both high heritability and high genetic advance are present, it indicates that additive gene action and phenotypic selection are likely to significantly enhance a specific trait. In the current study, it was found that 1000SW exhibited high heritability estimates along with significant genetic advance, suggesting minimal environmental influence^31,39^). This suggests that the specific trait is probably influenced by additive gene action, making direct phenotypic selection effective. Traits like the NPP and DTM exhibited high heritability with moderate genetic advance^29,40^). Conversely, SYP, CP, PB, SP, and PL displayed high heritability with low genetic advance^41,42^). In contrast, traits such as DTF, PH, and HI showed moderate heritability and limited genetic advance^43,44^) implying the involvement of non-additive gene action. Consequently, phenotypic selection in earlier generations may not yield promising outcomes for these traits.

### Correlation studies

**Fig. 1:**
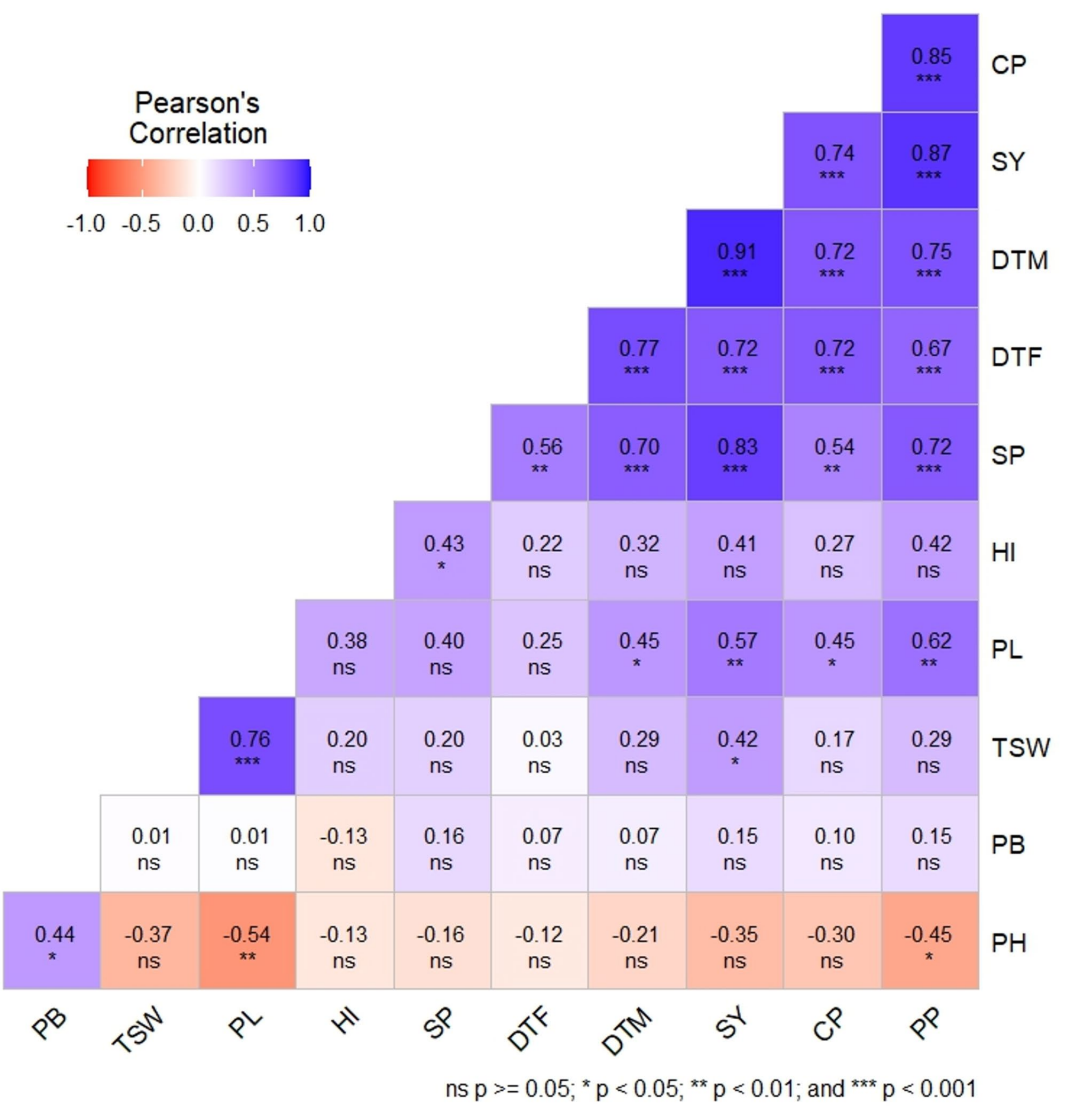
Correlation studies among different traits of horsegram.

Correlation analysis helps the plant breeder to understand the relative importance of different plant traits and provides a sufficient basis for selection. In this study, we studied correlation coefficients for 11 quantitative traits to determine their relative significance in influencing SY, a complex polygenic trait in plant breeding (Fig. 1).

Characters namely DTM, SY, CP, and NPP showed significant positive correlation with DTF^34^. This is helpful in developing early maturing varieties. In respect of DTF in blackgram^45^. DTM exhibited significant positive correlation with DTF, NPP, CP, and SY, which is helpful in the development of early maturing high-yielding varieties. Characters namely NPP, SP, DTM, DTF, CP, and PL correlated significantly positively with SY^46,47^). This suggests that improving any of these traits will lead to an improvement in SY. Traits including PL, SP, and TSW are showed significant positive correlation with SP^48^. These findings suggest that these traits play a supportive role in enhancing SY in horsegram.

**Table 3:** Analysis of variance for 11 morphological traits in 22 advanced breeding lines of horsegram.

Source of variation	d.f.	PH	PB	DTF	DTM	TSW	PL	SP	CP	NPP	HI	SY
Replication	2	16.25	1.483	11.10	7.81	0.50	0.031	0.005	0.93	4.57	10.52	0.093
Genotype	21	114.63**	2.89**	122.30**	228.24**	344.59**	0.47**	0.518**	55.66**	199.10**	85.76**	3.89**
Residuals	42	29.05	0.56	26.06	39.59	1.25	0.029	0.044	2.99	10.24	17.14	0.082

**Significance level at 1% level.

**Table 4:** Estimates of genetic parameters for yield attributing traits in 22 advanced breeding lines of horsegram.

S. No.	Characters	Range		Mean ± SE	GCV (%)	PCV (%)	Heritability (%)	Genetic advance	GAM
		Min.	Max.						
1.	PH	46.00	69.60	55.15 ± 3.11	9.68	13.75	49.54	7.74	14.03
2.	PB	4.71	8.36	6.311 ± 0.43	13.96	18.30	57.96	1.38	21.80
3.	DTF	53.67	77.67	63.78 ± 2.94	8.87	11.95	55.18	8.66	13.50
4.	DTM	96.67	125.33	110.81 ± 3.63	7.15	9.13	61.36	12.76	11.54
5.	TSW	22.57	54.34	37.82 ± 0.64	28.28	28.43	98.92	21.91	57.95
6.	PL	3.15	4.75	4.11 ± 0.09	9.34	10.24	83.25	0.72	17.56
7.	SP	4.02	5.68	4.56 ± 0.12	8.70	9.86	77.94	0.72	15.83
8.	CP	10.07	22.79	16.13 ± 0.99	25.97	28.10	85.43	7.97	49.45
9.	PP	18.22	57.23	32.89 ± 1.4	24.11	26.00	86.00	15.15	7.44
10.	HI	34.54	51.13	42.41 ± 2.39	11.27	14.91	57.16	7.44	17.56
11.	SY	3.32	7.58	4.74 ± 0.16	23.73	24.48	93.91	2.24	47.37

### D^2^ studies

**Fig. 2:**
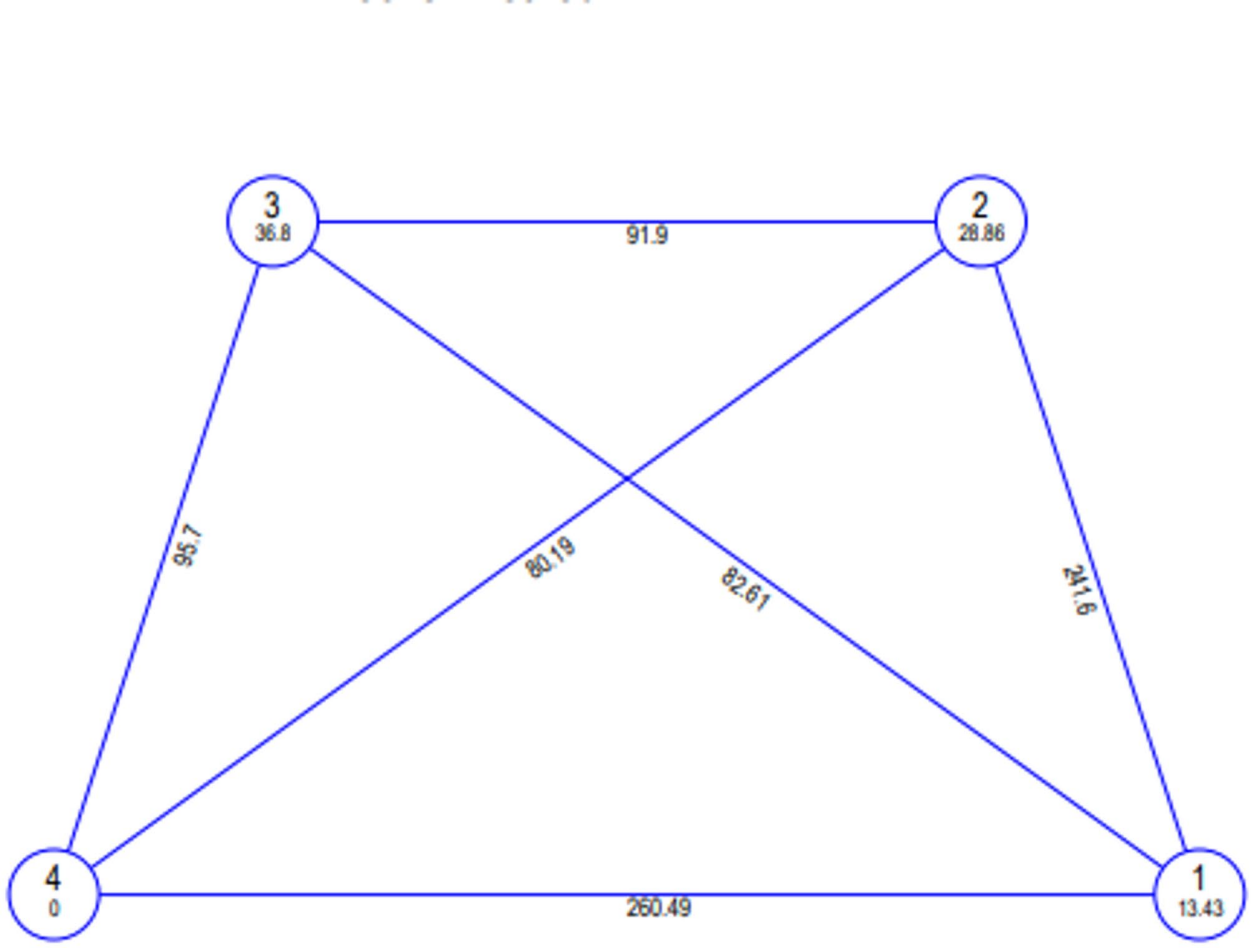
Mahalanobis Euclidean Distance of clusters through inter and intra cluster distribution in twenty-two genotypes.

**Fig. 3:**
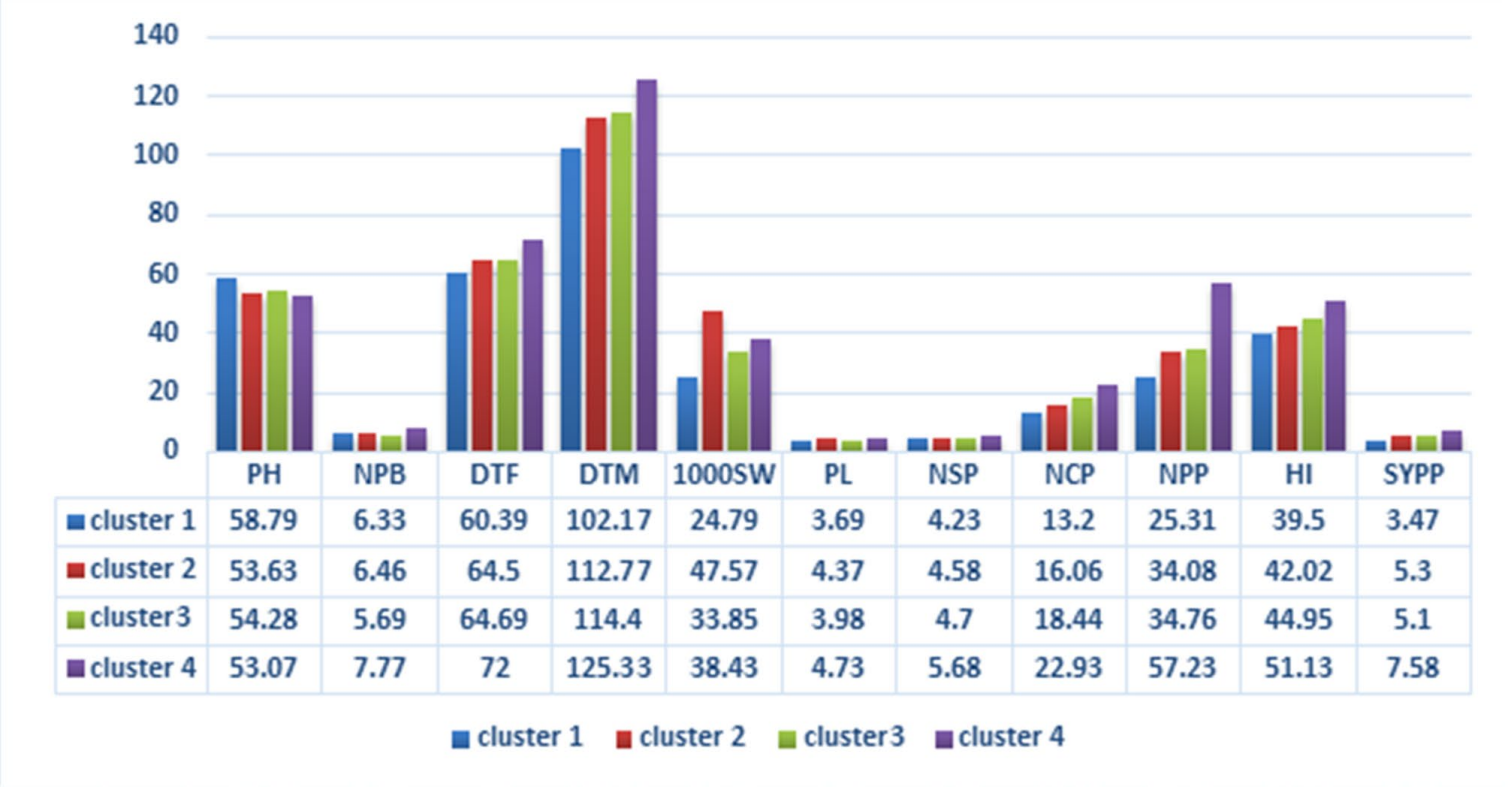
Mean performances of clusters for yield attributing traits of 22 advanced breeding lines of horsegram.

Multivariate analysis employing Mahalanobis D^2^ statistics stands as a well-established method for assessing genetic divergence, crucial for improvement programs in pinpointing suitable parent combinations to achieve valuable recombinant outcomes. In the current study, 22 selected genotypes underwent Mahalanobis D^2^ analysis, focusing on 11 traits. Employing Tocher’s clustering method, these 22 genotypes were categorized into four distinct clusters, as indicated in Table 5. Among these clusters, Cluster II was the most extensive, consisting of ten genotypes, while Cluster I contained six genotypes, Cluster III had five genotypes, and Cluster IV was the only isolated cluster. The study also computed average inter-cluster and intra-cluster distances based on the total D^2^ values, and the findings are detailed in Fig. 2. The highest inter-cluster distance was recorded between clusters I and IV (260.49), resulting in maximum divergence, followed by clusters I and II (241.60). The highest intra-cluster distance was noticed for cluster III (36.80) followed by cluster II (28.86), which showed that the genotypes present in the same cluster exhibit significant variability among themselves. Based on the cluster means (Fig. 3), cluster I demonstrated the highest mean performance for PH (58.79). On the other hand, cluster II had the highest mean performance for 1000 seed weight (47.57). Cluster IV, however, exhibited high mean performance for several traits, including the PB, DTF (72.00), DTM (125.33), PL (4.73), SP (5.68), CP (22.93), NPP (57.23), HI (51.23), and SY (7.58).

**Fig. 4:**
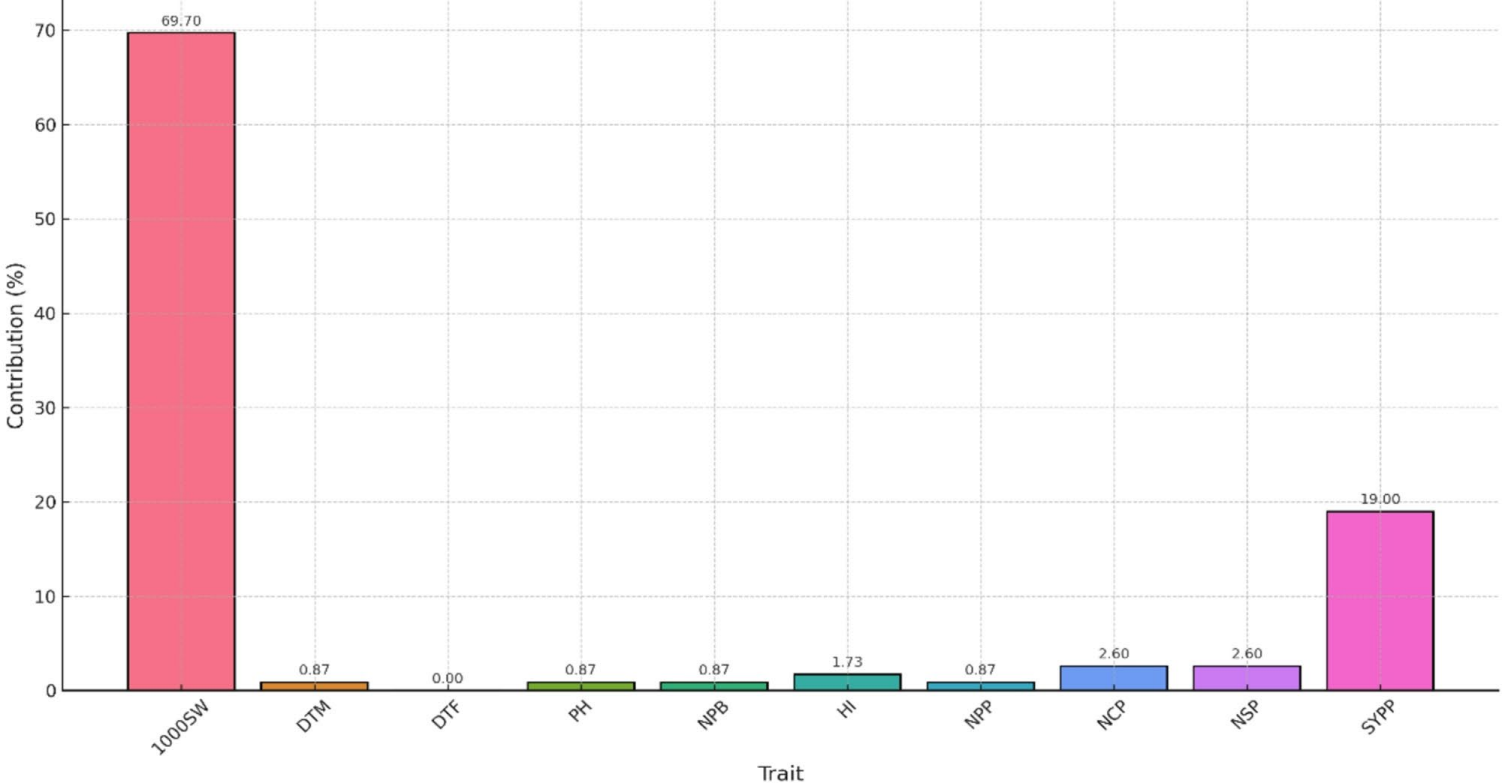
Contribution (%) of individual character towards divergence.

Considering Cluster IV’s superior mean performance in yield-related traits, advanced breeding lines from this cluster have potential as valuable candidates for horsegram hybridization improvement programs. When the relative contribution of each character towards divergence was calculated (Fig. 4), it was observed that 1000 SW (69.7%) contributed the most towards genetic divergence^49^. This was followed by SY (19.0%), SP, CP (2.6%), HI (1.73%), PH, PB, DTM, and PL (0.87%). Traits such as SY, PH, PB, NPP, SP, and DTM were also found to be influential factors contributing towards genetic divergence^20^.

**Table 5:** Grouping of twenty-two advanced breeding lines into four different clusters on the basis of D^2^ analysis.

Clusters	Number of lines	Lines/genotypes
I	6	AK-21, BSP-17-1, BSP-17-3, BSP-19-2, BSP-19-3, Thaira Kulthi-02
II	10	HPKC-11-74, MC-5-3, HPKC-11-47, HPKC-11-01, HPKC-11-57, MC-6-5, HPKC-11-25, HPKC-11-04, HPK-4, HPKC-02
III	5	Bilas9, HPKC-11-17, HPKC-11-56, MC-6-4, VLG-15
IV	1	HPKM-150

### Molecular analysis

**Fig. 5:**
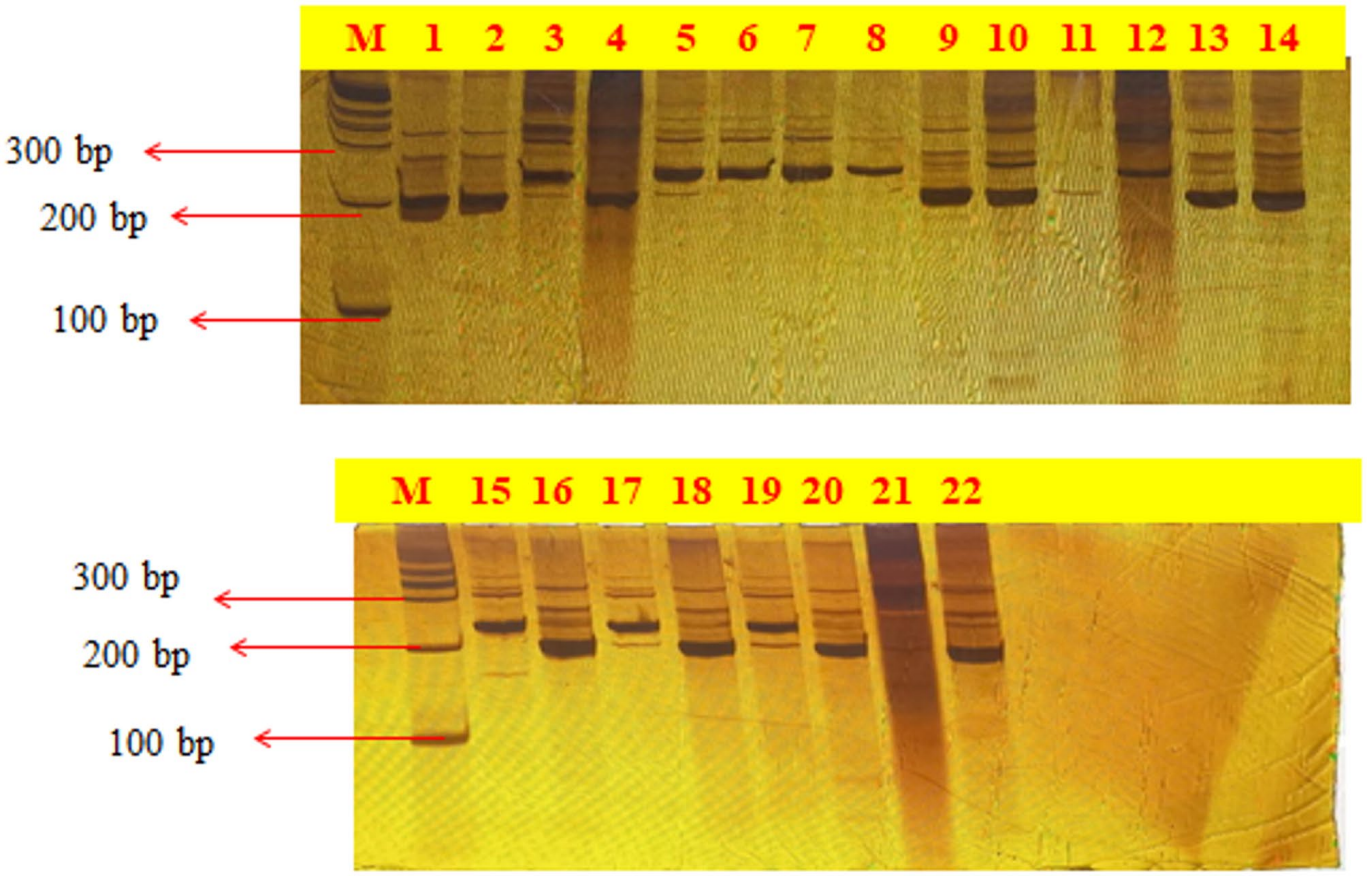
Pictorial representation of amplified PCR product of MUMS-24 marker on PAGE.

The diversity of horsegram has largely remained unexplored, with only a few studies focusing on the analysis of morphological and biochemical traits. The major hindrance in advancing the genetic improvement of horsegram has been the limited usage of genetic and genomic information and resources. Molecular markers are a reliable and accurate way to measure genetic diversity in germplasm. In this study, we used 30 SSR primers to characterize 22 horsegram genotypes. Out of 30 SSR primers, 09 were identified as polymorphic, namely, MUMS-18, MUMS-02, HUGMS-17, HTSSR-153, MUMS-35, MUMS-19, MUMS-24 (Fig. 5), HTSSR-151, and MUMS-7. PIC value, number of alleles and resolving power of SSR primers are represented in Table 6. A total of 23 alleles were identified with an average of 2.56 alleles per locus, ranging from 2 to 4 across the lines. Average PIC (2.56) in the current study is comparable to values observed in horsegram^4,50^), where average PIC values of 2.64 and 2.6 were recorded using SSR primers. In blackgram, PIC values ranging from 0.18 to 0.70 were observed^51^. MUMS-02 amplified the maximum number of alleles (4), followed by HUGMS-17, HTSSR-151, and HTSSR-153 with 3 alleles each, while MUMS-7, MUMS-18, MUMS-19, and MUMS-35 identified 2 alleles. The PIC values ranged from 0.09 to 0.70. The highest PIC was identified in MUMS-18 (0.70), followed by MUMS-02 (0.64), HUGMS-17 (0.62), and HTSSR-153 (0.61). The lowest PIC value was observed in MUMS-7 (0.09). Further, the polymorphism revealed by the SSR markers used in the current study is comparable to findings in horsegram^2,19^). SSR markers also play an important role in molecular studies of pulses^52^.

**Fig. 6:**
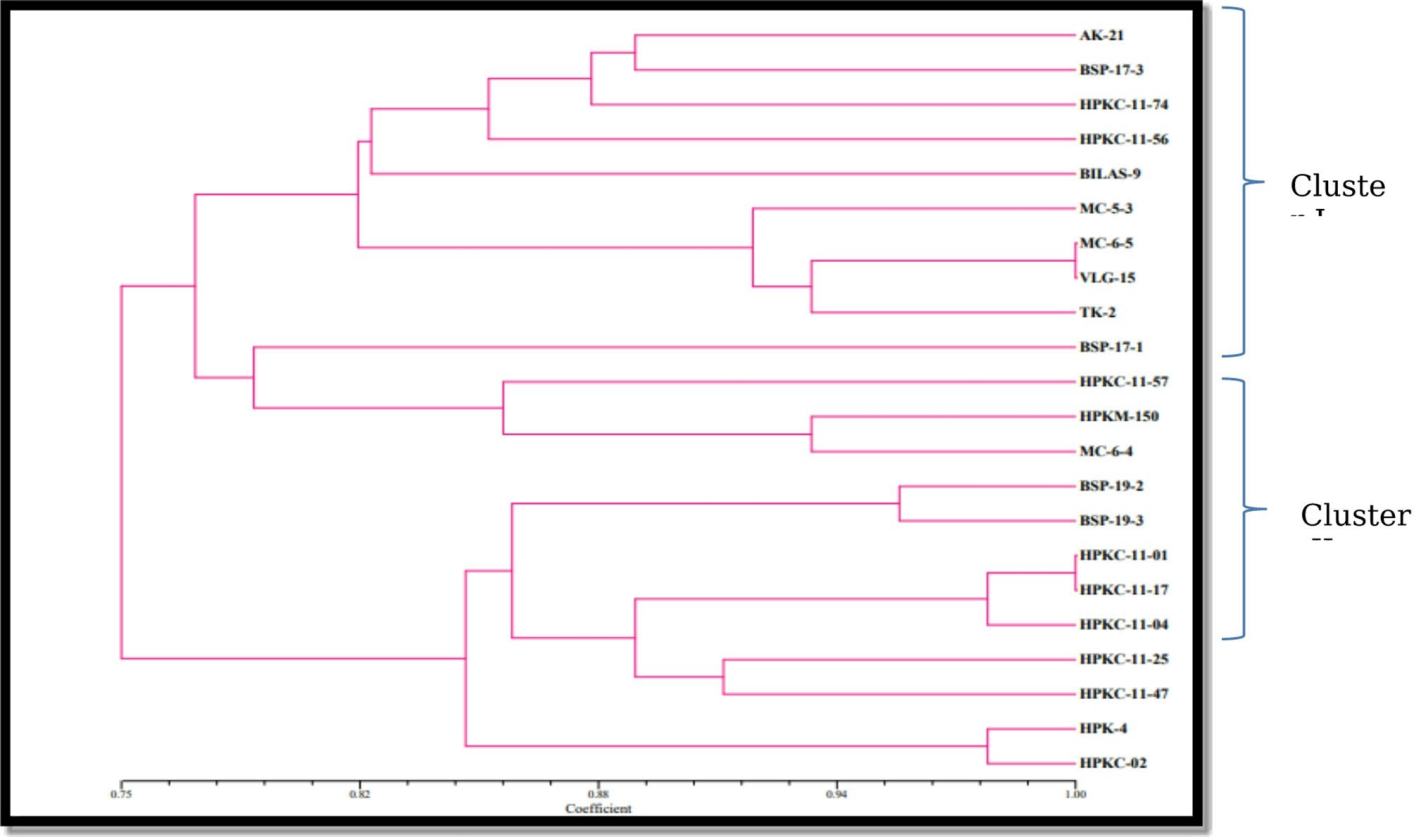
Details of grouping of horsegram genotypes into different clusters based on Jaccards similarity coefficient.

**Fig. 7:**
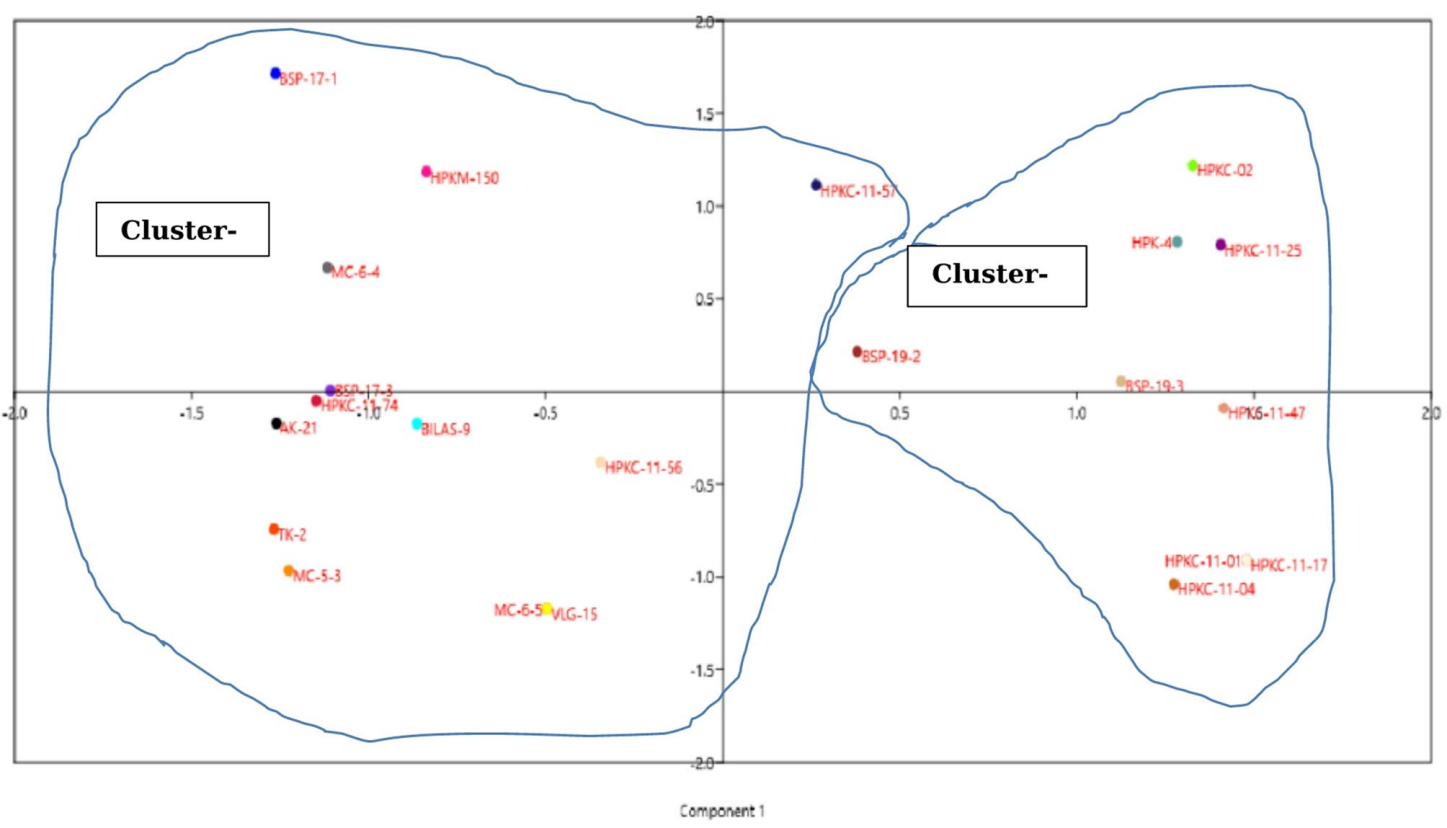
Principal component analysis (PCA) of 22 horsegram genotypes by 30 SSR primers.

Dendrogram based on Jaccards similarity coefficient and UPGMA method showed major two groups as shown in Fig. 6 with coefficient values ranging from 0.6 to 0.95, indicating varying degrees of similarity between the lines. Group-I comprised of nine genotypes/lines. This was further divided into sub-groups I-a and I-b. Group-II comprised of 13 genotypes/lines and was further divided into sub-groups II-a and II-b. The cluster analysis, encountered two ties, between lines HPKC-11-01 and HPKC-11-17, and between lines MC-6-5 and VLG-15. These ties indicate the presence of maximum similarity between the respective pairs of lines. Two dimensional representations of data by PCA also showed the two major groups (Fig. 7). Clustering patterns of PCA complemented the clustering of the dendrogram based on Jaccard’s similarity coefficient and UPGMA, aligning with findings in horsegram^11,37,38^).

**Table 6:** Details of polymorphic SSR primers along with the number of alleles and polymorphism information content (PIC).

S. No.	Primer name	No. of alleles	PIC value
1.	HUGMS-17	3	0.62
2.	HTSSR-151	3	0.22
3.	MUMS-7	2	0.09
4.	MUMS-18	2	0.70
5.	MUMS-19	2	0.50
6.	HTSSR-153	3	0.61
7.	MUMS-24	2	0.48
8.	MUMS-35	2	0.50
9.	MUMS-02	4	0.64

## Conclusion

This investigation effectively integrated molecular, and morphological studies, to provide a comprehensive understanding of the genetic diversity among horsegram genotypes. Morphological data revealed significant variability, with traits like 1000SW, SY, and NPP exhibiting high heritability and genetic advance, indicating their suitability for phenotypic selection at early stage. Correlation analysis demonstrated strong positive associations between traits such as the NPP, DTM, and 1000SW with SY, highlighting their significance in the development of high-yielding breeding lines or varieties. Molecular analysis using SSR markers supported these findings by identifying sufficient amount of genetic diversity, with UPGMA and PCA clustering complementing the morphological and correlation-based groupings. This congruence between molecular, and morphological studies highlights the reliability of the results and provides a robust framework for targeted breeding programs aimed at improving seed yield-related traits in horsegram and also showed sufficient amount of genetic diversity at phenotypic as well as molecular level.

## Data Availability

All data generated or analyzed during this study are included directly in the text of this submitted manuscript. There are no additional external files with data.

## Abbreviations

d.f.: Degrees of freedom
PH: Plant height (cm)
PB: No. of primary branches
DTF: Days to 50% flowering
DTM: Days to maturity
1000SW: 1000 Seed weight (g)
PL: Pod length (cm)
SP: No. of seed per pod
CP: No. of cluster per plant
NPP: No. of pod per plant
HI: Harvest index (%)
SY: Seed yield per plant (g)
